# Toward Sub‐Terahertz: Space‐Time Coding Metasurface Transmitter for Wideband Wireless Communications

**DOI:** 10.1002/advs.202304278

**Published:** 2023-08-08

**Authors:** Yujie Liu, Yu Wang, Xiaojian Fu, Lei Shi, Fei Yang, Jiang Luo, Qun Yan Zhou, Yuan Fu, Qi Chen, Jun Yan Dai, Lei Zhang, Qiang Cheng, Tie Jun Cui

**Affiliations:** ^1^ State Key Laboratory of Millimeter Waves Southeast University Nanjing 210096 China; ^2^ Institute of Electromagnetic Space Southeast University Nanjing 210096 China; ^3^ School of Electronics and Information Hangzhou Dianzi University Hangzhou 310018 China

**Keywords:** metasurface, space‐time coding, sub‐terahertz, wireless communications

## Abstract

A space‐time coding metasurface (STCM) operating in the sub‐terahertz band to construct new‐architecture wireless communication systems is proposed. Specifically, a programmable STCM is designed with varactor‐diode‐tuned metasurface elements, enabling precise regulation of harmonic amplitudes and phases by adjusting the time delay and duty cycle of square‐wave modulation signal loaded on the varactor diodes. Independent electromagnetic (EM) regulations in the space and time domains are achieved by STCM to realize flexible beam manipulations and information modulations. Based on these features, a sub‐terahertz wireless communication link is constructed by employing STCM as a transmitter. Experimental results demonstrate that the STCM supports multiple modulation schemes including frequency‐shift keying, phase‐shift keying, and quadrature amplitude modulations in a wide frequency band. It is also shown that the STCM is capable of realizing wide‐angle beam scanning in the range of ±45^o^, which offers an opportunity for user tracking during the communication. Thus, the STCM transmitter with high device density and low power consumption can provide low‐complexity, low‐cost, low‐power, and low‐heat solutions for building the next‐generation wireless communication systems in the sub‐terahertz frequency and even terahertz band.

## Graphical abstract

In this work, a new‐architecture sub‐terahertz transmitter for wideband wireless communication applications based on STCM is reported. These experimental findings validate that the STCM is capable of supporting multiple modulation schemes of the digital information and wide‐angle beam scanning of sub‐terahertz waves. STCM‐based communication transmitters can provide a low‐complexity, low‐cost, and power‐efficient approach to future wireless communication systems.

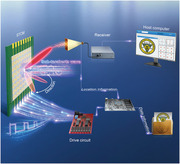

## 1 Introduction

Next‐generation wireless communication systems are expected to achieve unprecedented communication rates of Tbps, ultra‐low latency of sub‐milliseconds, greater reliability, denser connectivity, and support for extremely precise positioning, immersive interactive experiences, and multi‐dimensional perception.^[^ ^1^ ^]^ To meet these demands, ample spectrum resources are required, which cannot be satisfied by the existing communication bands, necessitating an expansion toward terahertz bands with rich spectrum resources.^[^ ^2^, ^3^, ^4^ ^]^ The sub‐terahertz band has been proposed for communication applications recently, and new radio technologies have been developed in 90–200 GHz with performance beyond 5G.^[^ ^5^, ^6^, ^7^ ^]^ However, conventional wireless communication transmitters typically consist of signal modulation, digital‐to‐analog conversion, mixing, radiation, and other essential parts.^[^ ^8^ ^]^ Designing communication systems based on the conventional technology in the sub‐terahertz band requires numerous high‐performance radio frequency (RF) links and antennas, which makes the communication system both complex and expensive.^[^ ^9^ ^]^ Moreover, large free‐space attenuation in the sub‐terahertz band necessitates a highly directional beam to reduce aggregated co‐channel interference and severe propagation loss.^[^ ^7^, ^10^ ^]^ This poses a challenge for signal coverage in sub‐terahertz communication. Thus a cost‐effective solution with wide signal coverage for sub‐terahertz communication systems is urgently needed.

Metasurface shines a new light on the architecture of the sub‐terahertz communication transmitter. The metasurface is a 2D variant of metamaterial, typically comprising arrays of subwavelength artificial elements with deep subwavelength thicknesses.^[^ ^11^, ^12^, ^13^ ^]^ Its unique properties that allow for flexible manipulations of electromagnetic (EM) waves generate widespread interest in the scientific community. Recently, digital coding metasurfaces and programmable metasurfaces have been successively developed, forging a connection between EM physics and digital information.^[^ ^14^, ^15^, ^16^, ^17^ ^]^ This has led to the realization of a range of fascinating applications such as anomalous refraction and reflection of EM beams,^[^ ^18^, ^19^ ^]^ dynamic beamforming,^[^ ^20^, ^21^, ^22^ ^]^ polarization modulation,^[^ ^23^, ^24^ ^]^ vortex beam generation,^[^ ^25^, ^26^ ^]^ and holographic imaging.^[^ ^27^, ^28^, ^29^ ^]^ Subsequently, time‐domain digital coding metasurfaces were proposed, and hence the manipulation of EM waves is extended from the space domain to the time domain, and the functionalities of metasurfaces are greatly expanded,^[^ ^30^, ^31^ ^]^ including nonlinear harmonic regulation,^[^ ^32^ ^]^ efficient frequency synthesis,^[^ ^33^ ^]^ multi‐polarization conversion,^[^ ^34^ ^]^ and nonlinear convolution operations.^[^ ^35^ ^]^ Furthermore, space‐time coding metasurface (STCM) combines the encoding of EM characteristics in both time and space domains.^[^ ^36^ ^]^ This allows for the simultaneous controls of wave propagation direction in the space domain and the manipulation of spectral distribution in the frequency domain,^[^ ^37^ ^]^ and integrates richer functions in a single device.^[^ ^38^ ^]^

Now the programmable metasurface has emerged as a promising technology for wireless communications. By manipulating the propagation path of EM waves, the metasurface can reconfigure the wireless environment to solve the signal coverage problems.^[^ ^39^, ^40^, ^41^ ^]^ The time‐domain coding metasurface offers the ability to modulate the EM spectra in real time, enabling low‐cost wireless communication transmitters.^[^ ^42^, ^43^, ^44^, ^45^ ^]^ Definitely, the current attempts to apply for the time‐domain coding metasurfaces in communication systems have been focused primarily on low and medium frequency bands. In the millimeter‐wave band ≈28 GHz, space‐domain coding metasurface and time‐domain coding metasurface have been investigated for communication transmitters and beamforming.^[^ ^44^, ^46^ ^]^ However, the metasurface‐based wideband communication system capable of implementing multiple modulation schemes while integrating the information modulation and beamforming simultaneously is rarely reported. The STCM can present an opportunity to develop a novel wireless communication transmitter that seamlessly integrates beam steering and information modulation, enabling an active, intelligent, and controllable wireless communication environment.

Additionally, we remark that significant advancements have been achieved in manipulating terahertz waves using metasurfaces.^[^ ^47^, ^48^ ^]^ Several noteworthy outcomes have been reported regarding the modulations of the terahertz waves using metasurfaces.^[^ ^49^, ^50^ ^]^ However, the application of metasurfaces for space information modulations in the terahertz frequencies, particularly in the context of communications, and the integration of information modulation with beam steering, have not been extensively explored. This is primarily attributed to the fact that the programmable metasurfaces designed for terahertz‐wave regulations typically rely on tunable materials, such as liquid crystals^[^ ^51^ ^]^ and vanadium dioxide.^[^ ^52^, ^53^ ^]^ These materials face some challenges in the terahertz frequencies, including long response time and complex control, which inevitably increase the difficulty of terahertz time‐domain modulation. Moreover, the substantial propagation loss in the sub‐terahertz necessitates the use of directional beams for precise user tracking during communications.^[^ ^4^, ^54^ ^]^ Therefore, the development of sub‐terahertz STCM capable of efficient information modulation and beam scanning is urgently needed to realize cost‐effective and efficient wireless communications in the next‐generation systems.

**Figure 1:**
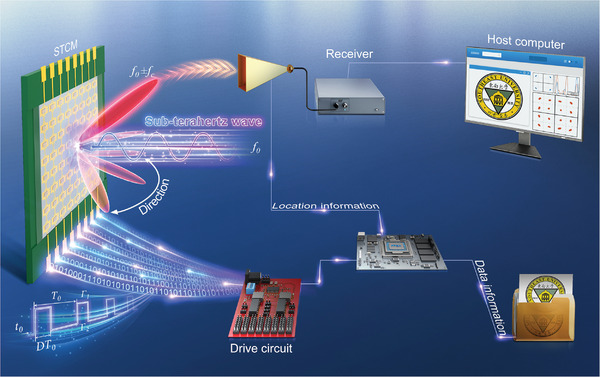
The conceptual diagram of the sub‐terahertz wireless communication system based on the STCM transmitter. The data intended for transmission are mapped into the control signals for STCM through a control module composed of a field programmable gate array (FPGA) and driver circuitry. By precisely controlling the varactor diodes in different STCM units, which is enabled by control signals composed of square waves with varying time delays and duty cycles, we can achieve accurate modulations of amplitudes and phases of the reflection harmonics, thereby integrating the information modulation and beamforming. The incident sub‐terahertz waves can be modulated by STCM using different modulation schemes, such as FSK, PSK, and QAM, while beam steering is simultaneously achieved. The receiver processes the modulated sub‐terahertz signals and reconstructs the original data information, which is then displayed on the host computer.

Here, we present an STCM operating in the sub‐terahertz frequency range, along with a space‐time domain‐separated coding method. This method, which is based on independent and precise controls of harmonic phases and amplitudes, enables the integration of communication transmitters and beam steering systems. We design a 1‐bit phase coding metasurface and employ reverse‐biased varactor diodes as the phase‐shifting components in STCM to effectively avoid the generation of excessive heat and high power consumption that occur in high‐density STCM. Subsequently, a sophisticated method is presented to control the reflection amplitudes and phases of harmonics by precisely adjusting the time delay and duty cycle between the switching periods of different units. Afterward, we propose a straightforward space‐time uncoupled coding method that achieves independent controls in both space and time domains. Accordingly, the communication transmitter and beam scanning can be simultaneously implemented with the STCM. The accurately computed time‐domain coding allows for realizing various modulation schemes such as frequency shift keying (FSK), phase shift keying (PSK), and quadrature amplitude modulation (QAM), demonstrating the versatile and robust modulation capabilities of the designed STCM transmitter. Finally, a broadband sub‐terahertz wireless communication system is constructed by employing the STCM transmitter. The simplified conceptual diagram of the wireless communication system is shown in **Figure** **1**, which operates in a wide frequency band (81.25–94.65 GHz) and effectively implements a range of modulation schemes, with the optimal results at 88 GHz. We also experimentally demonstrate that the system produces beamforming and beam scanning performance within ±45^o^, which is sufficient for effective user tracking in most scenarios and well suited for narrow‐beam directional communications in the sub‐terahertz frequencies. The proposed STCM offers low‐complexity, cost‐effective, low‐power, and low‐heat solutions for constructing an intelligently controlled wireless propagation environment in the sub‐terahertz frequency, catering to the requirements of next‐generation wireless communication systems.

## 2 Design of STCM in the Sub‐Terahertz Frequency

**Figure 2:**
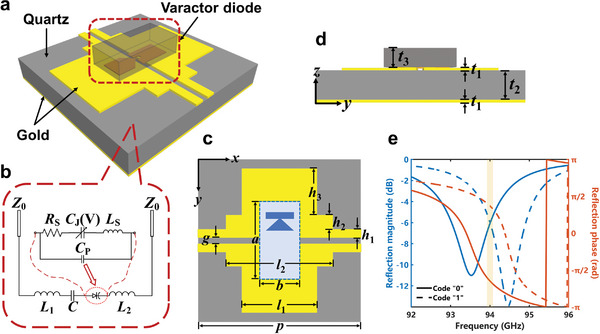
a) Perspective view of the STCM unit operating at 94 GHz, which consists of three distinct layers. b) The equivalent circuit of the unit structure. c) The top layer of the STCM unit is etched with two symmetrical stepped gold patches with flip‐chip varactor diodes attached to them. As seen from (d) the side view, the middle layer is a quartz substrate, with its bottom covered by a gold film. e) S11 simulated results of the STCM unit when the bias voltage is 0 (code “0”) and 24 V (code “1”), respectively.

We design STCM to operate at a target frequency of 94 GHz, with its unit structure illustrated in **Figure** **2**a. The unit structure comprises three distinct layers, in which the bottom of the quartz substrate is covered with gold film to reflect the EM waves, while two symmetrical stepped metal patches are etched onto its top surface. A flip‐chip varactor diode is centrally attached between the two metal patches, serving as a tuning element. The two metal patches are connected to the DC signal and ground, respectively. The equivalent circuit of the STCM is depicted in Figure 2b. As the bias voltage applied across the varactor diode is altered, its capacitance will change, which leads to the tunable reflection property of the STCM unit (see Note S1, Supporting Information, for more details).

The detailed geometric design is presented in Figure 2c,d, with the following parameters: *p* = 1500 µm, *l*_1_ = 690 µm, *l*_2_ = 980 µm, *h*_1_ = 77.5 µm, *h*_2_ = 130 µm, *h*_3_ = 425 µm, *g* = 55 µm, *a* = 665 µm, *b* = 340 µm, *t*_1_ = 0.3 µm, *t*_2_ = 200 µm, and *t*_3_ = 175 µm. The flip‐chip varactor diode employed in this design is MACOM's MA46H146, which supports a reverse bias voltage of up to 25 V. Simulation results indicate that the reflected phase of the unit corresponds to *θ* (code “0”) when the bias voltage is 0; while the reflected phase of the unit corresponds to *θ* + *π* (code “1”) when the bias voltage is 24 V, as detailed in Note S2 (Supporting Information). From Figure 2e, we note that the phase difference between the two states reaches *π* at 94 GHz, with an identical reflection loss of 6 dB. On this basis, the optimized unit structure can be used to design the 1‐bit STCM. To further validate the STCM's ability to manipulate the EM waves, we arrange the STCM in a specific coding state. Owing to the constraints in processing and components, we can only independently control the STCM units column by column. As depicted in Figure 2c, the coding varies along the *y*‐direction. In accordance with the generalized Snell's law of reflection,^[^ ^18^ ^]^ the direction of the reflected beam is determined by the phase gradient across the metasurface. When the EM wave is normally incident on the STCM from the air, the following equation holds

(1) $$\theta_{r}=\arcsin\left(\frac{\lambda }{2\pi n_{i}}\frac{d\phi }{dy}\right)$$

where *θ*_*r*_ denotes the reflection angle, *λ* represents the working wavelength, *n_i_* indicates the refractive index of air, and *d* *ϕ*/*dy* signifies the phase gradient along the *y*‐axis. Consequently, the required phase *ϕ*_*m*_ for the *m‐*th column of the STCM can be expressed as

(2) $$\phi_{m}=-\frac{2\pi n_{i}}{\lambda }md_{y}\sin\theta_{r}+\phi_{0}$$

where *d_y_* is the period length of the STCM unit along the *y*‐direction and *ϕ*_0_ is the initial phase. From Equation (1), the reflection angle *θ*_*r*_ is not related to *ϕ*_0_; thus, we let *ϕ*_0_ = 0. Quantization of the continuous values calculated from Equation (2) results in the subsequent equation

(3) $$\mathrm{code}\left(m\right)=\left\{\begin{array}{cc} 0, & 0\leq \phi_{m}<\pi \\ 1, & \pi \leq \phi_{m}<2\pi \end{array}\right.$$

Based on this, we can compute the coding states for a specific reflection angle, facilitating dynamic beamforming. As the coding metasurface manipulates the EM waves solely in the space domain at this stage, we refer to it as space‐domain coding. We design the metasurface with a variety of coding states and numerically simulate the far‐field scattering patterns of the metasurface. The results demonstrate that the designed STCM is capable of achieving beam scanning within the ±55^o^ range (see Note S3, Supporting Information, for more details). This indicates that the STCM possesses dynamic beamforming capabilities, laying the groundwork for further employing the time‐domain coding to control STCM and construct the STCM communication transmitter and beam scanning system.

## 3 Manipulation of Harmonics by Time‐Domain Coding

The proposed STCM can control its reflection phase in the sub‐terahertz range by tuning the voltage applied to the varactor diode. By controlling the bias voltage using an FPGA and drive circuit in a time‐varying manner, the reflection phase of the STCM will also vary over time. This enables dynamic controls over each order harmonic component of the reflected wave using the methods described below.

*Reflection coefficient in the time domain*: The STCM unit can generate two distinct reflection coefficients, denoted as $\Gamma_{1}=A_{1}e^{j\varphi_{1}}$ and $\Gamma_{2}=A_{2}e^{j\varphi_{2}}$, where *A*_1_ and *A*_2_ denote the reflection amplitudes, while φ_1_ and φ_2_ represent the reflection phases. Thus, the reflection coefficient Γ(*t*) of the metasurface can be seen as a periodic square wave with two states Γ_1_ and Γ_2_, which could be expressed as

(4) $$\Gamma \left(t\right)=\Gamma_{2}+\left(\Gamma_{1}-\Gamma_{2}\right)\cdot u\left(t-t_{0}\right)$$

where *t*_0_ is the time delay and *u*(*t*) is a square‐wave function with period *T*_0_ and duty cycle *D*. Γ(*t*) can further be represented as a Fourier series as follows

(5) $$\Gamma \left(t\right)=\Gamma_{2}+\sum_{n=-\infty }^{+\infty }a_{n}e^{\mathrm{jn}\omega_{0}\left(t-t_{0}\right)}$$

in which *a_n_* = *h* *DSa*(*n*π*D*), ω_0_ = 2π*f*_0_, and *h* depends on the two states of the square‐wave signal *h* = Γ_1_ − Γ_2_=|*h*|*e* ^*j* *φ*^_0_.

*Reflection coefficient in the frequency domain*: To analyze the characteristics of harmonics more intuitively, we rewrite the reflection coefficient in the frequency domain

(6) $$\Gamma \left(f\right)=\Gamma_{2}\delta \left(f\right)+\sum_{n=-\infty }^{+\infty }a_{n}e^{-\mathrm{jn}\omega_{0}t_{0}}\delta \left(f-nf_{0}\right)$$

*Reflected wave in the frequency domain*: Suppose that *E_i_* (*f*) is a monochromatic plane wave with the frequency of *f_c_*. Then the reflected wave *E_r_* (*f*) can be expressed as

(7) $$E_{r}\left(f\right)=\Gamma \left(f-f_{c}\right)=\Gamma_{2}\delta \left(f-f_{c}\right)+\sum_{n=-\infty }^{+\infty }a_{n}e^{-\mathrm{jn}\omega_{0}t_{0}}\delta \left(f-f_{c}-nf_{0}\right)$$

*Amplitude and phase coefficients*: According to Equation (7), the amplitude and phase of the reflected harmonic are influenced by the difference between the two states of the square‐wave signal, the duty cycle, and the time delay. The amplitude and phase coefficients of the *n‐*th harmonic can be written as

(8) $$\begin{array}{c} A=\left|h\right|\mathrm{DSa}\left(n\pi D\right),0\leq D\leq 1/2\left|n\right|\\ \varphi =\varphi_{0}-n\omega_{0}t_{0},0\leq t_{0}<T_{0}/\left|n\right| \end{array}$$

Thus, by maintaining the two states of the square‐wave signal constant, we are able to control the amplitude and phase of the harmonics through the manipulation of the duty cycle *D* and the time delay *t*_0_. Notably, as demonstrated by Equation (8), these two parameters exhibit no coupling, which facilitates the straightforward regulation of the amplitude and phase of the reflected harmonic by employing the direct output of the square wave generated by the FPGA and drive circuit.

**Figure 3:**
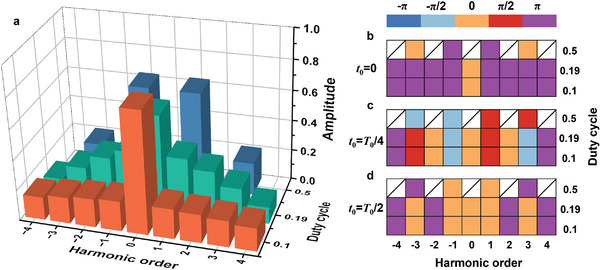
The modulation relationship of square wave modulation signals with different duty cycles and different time delays to the amplitude and phase of harmonics. a) Square wave signals with different duty cycles will result in different harmonic amplitude distributions. Harmonic phase distributions under the control of b) *t*_0_ = 0, c) *t*_0_ = *T*_0_/4, and d) *t*_0_ = *T*_0_/2 square wave signals, respectively.

Owing to the constraints of fabrication process and active components, designing a multi‐bit metasurface in the sub‐terahertz frequency presents a significant challenge, limiting the flexibility of the coding metasurface for manipulating EM waves. However, by incorporating time delays, it is possible to transform the 1‐bit metasurface into STCM capable of operating in multi‐bit by exploiting the harmonics. Simultaneously, harmonic amplitude can be modulated by controlling the duty cycle. To clarify the modulation relationship between the time delay and duty cycle of the square‐wave modulation signal on the phases and amplitudes of the harmonics, calculations are conducted under the conditions of *A*_1_ = *A*_2_ = 1, φ_1_ = π, and φ_2_ = 0, as illustrated in **Figure** **3**. As shown in Figure 3a, when the duty cycle *D* varies from 0.5 to 0.1, the amplitude distribution of the harmonics changes, while the phase distribution remains constant or exhibits a phase difference of π. In contrast, when the time delay *t*_0_ shifts from 0 to *T*_0_/2, the amplitude distribution of the harmonics stays consistent, whereas the phase distribution changes, which is demonstrated in Figure 3b–d. This observation highlights the simplicity of controlling the phase and amplitude of harmonics by adjusting the time delay and duty cycle of the modulation signal.

To validate the proposed harmonic control approach, a 16 × 8 STCM sample was fabricated based on the design process. The stepped metal patch and electrodes connected to the external control circuit were produced on the quartz substrate using a photolithographic technique. The varactor diode was secured at the designated position on the metal patches through the conductive silver paste. The metal backplane on the bottom was also created via photolithography. The metal electrodes of each sub‐array were connected to the PCB using conductive tape, and control signals were applied through pads on the PCB. Due to limitations in the processing and fabrication procedures, the actual fabricated samples function optimally at 88 GHz (see Note S4, Supporting Information, for more details).

**Figure 4:**
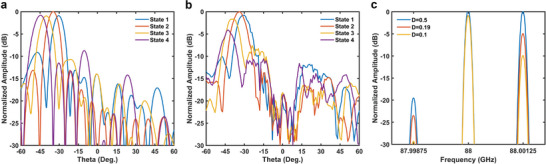
a,b) Calculations and experiments are performed by controlling the +1 harmonic phase by altering the time delay of the square‐wave control signal, enabling the beam deflections, in which Coding State 1 (1123 0123 0112 3012), Coding State 2 (0123 0123 0123 0123), Coding State 3 (0123 0120 1230 1201), and Coding State 4 (0123 1230 2301 3012) correspond to −30°, −35°, −40°, and −45°, respectively. c) The experimental results in modulating the harmonic amplitudes by changing the duty cycle of the modulation signal.

Considering the aforementioned scenario, further validation was conducted by performing the +1st order harmonic beam steering experiments at the frequency of 88 GHz using STCM. Four distinct modulation signals, with a frequency of 1.25 MHz, a duty cycle of 0.5, and time delays of 0, − *T*_0_/4, − 2*T*_0_/4, and − 3*T*_0_/4, were employed. Based on Equation (8) and assuming φ_0_ = 0, these signals can produce phase‐shifted states of 0, π/2, π, and 3π/2 for the +1st order harmonics, respectively, while maintaining a constant amplitude. This result is consistent with the characteristics of a 2‐bit coding metasurface. The four time‐delay signals can be encoded as “0”, “1”, “2”, and “3”, respectively. Therefore, we calculated the coding states for specific deflection angles in a manner similar to Equations (2) and (3), and simulated and measured the far‐field scattering patterns. The results are displayed in **Figure** **4**a,b. As the time‐domain approach at this stage enables the STCM to achieve the response of only four phase states, we also manipulate the +1st order harmonic in the space‐domain using solely these four phase states. This process is still a space‐domain coding for the +1st order harmonic, except that the four‐phase states are generated by switching the time delay signal, an effect akin to that produced by regulated voltage. Due to the tuning of the harmonic phase, the deflection angle of the main lobe in the measured far‐field pattern aligns well with the simulated outcomes. To further validate the control of the harmonic amplitude by the modulation signal, we selected the coding states with a deflection angle of −35^o^, altered the duty cycle of the signal, and measured its spectrogram, as illustrated in Figure 4c. According to the results in **Figure** **3**, for the +1st order harmonics, we normalize the amplitude values and express them in dB. The gains for D = 0.5, 0.19, and 0.1 are ≈0, −5 dB, and −10 dB, respectively. The measured gains are 0, −4.97 dB, and −9.94 dB, respectively, indicating that the modulation signal can effectively regulate the harmonic amplitude. The harmonic phase and amplitude can be well controlled using the modulation signal with varying time delay and duty cycle, providing a solid foundation for the subsequent implementation of complex functions such as information modulation and beam scanning in the sub‐terahertz frequency range using the STCM.

## 4 Design of Sub‐Terahertz Wireless Communication Transmitter Based on STCM

In conventional wireless communication systems, the modulation process usually involves controlling the carrier‐wave parameters with the modulating signal, so that one or more carrier‐wave parameters will vary according to the modulating signal's pattern. The modulated carrier signal retains all the characteristics of the modulating signal. After frequency conversion to high frequency by the RF module, the signal can be transmitted via an antenna for data transmission. As discussed in the previous section, the frequency, phase, and amplitude of the reflected harmonics can be controlled by loading STCM with modulation signals of varying frequencies, time delays, and duty cycles. To facilitate the implementation of diverse modulation schemes, such as FSK, PSK, and QAM, we need only to ensure that the frequency, phase, and amplitude of the reflected harmonics change according to the specific time patterns. These time patterns are defined as time codings, which allow for the mapping of transmitted information to the corresponding time coding based on various modulation schemes while maintaining the phase gradient induced by space coding either unchanged or reversed.

To facilitate communication, it is crucial to establish a mapping between the data message symbols and the reflection coefficients Γ(*t*). We can decompose Γ(*t*) into the product of the complex reflection coefficient Γ_*m*_(*t*), derived from the mapping of message symbols, and the fundamental pulse‐shaping function *g*(*t*). The relationship can be expressed as follows

(9) $$\begin{array}{ccc} \Gamma \left(t\right)=\Gamma_{m}\left(t\right)g\left(t\right), & 0\leq t\leq T^{\prime }, & \Gamma_{m}\left(t\right)\in M \end{array}$$

where *T*′ represents the duration of a data symbol and *M* represents a constellation point set with a basis size of |*M*|. In wireless communication, each data symbol Γ_*m*_(*t*) carries log _2_|*M*|‐bit data. During the duration of a single data symbol, Γ_*m*_(*t*) = *Ae* ^*j*φ^,0 ≤ *t* ≤ *T*′. Additionally, as demonstrated in Figure (4), to achieve high modulation power efficiency, we conduct wireless communication using the 1st order harmonic.

For standard binary FSK (BFSK) modulations, the set of constellations is given by $\Gamma_{m}\left(t\right)=e^{j2\pi f_{m}t},f_{m}\in M=\left\{f_{1},f_{2}\right\}$, where *f*_1_ and *f*_2_ represent the required frequencies for BFSK modulation. According to Equation (1) and (8), and the experiments in Section 3, it is evident that the space coding of the +1st order harmonic results in a symmetrical −1st order harmonic with the incident direction. Consequently, by reversing the space coding of the +1st order harmonic at a specific angle, a −1st order harmonic can be obtained at the same angle (see Note S5, Supporting Information, for more details). For instance, the space coding “0123 0123 0123 0123” can yield a +1st order harmonic with a deflection angle of −35^o^, while its reverse space coding “3210‐ 3210 3210 3 210” will produce a −1st order harmonic at the same angle. Therefore, we can utilize the −1st order harmonic frequency as *f*_1_ and the +1st order harmonic frequency as *f*_2_. For more complex FSK communication, it can be implemented by altering the frequency of the modulation signal, enabling us to achieve FSK communication effectively.

For standard quadrature PSK (QPSK) modulations, the set of constellations is given by

(10) $$\Gamma_{m}\left(t\right)\in M=\left\{e^{j\frac{\pi }{4}},e^{j\frac{3\pi }{4}},e^{j\frac{5\pi }{4}},e^{j\frac{7\pi }{4}}\right\},\left|M\right|=4,m=0,1,2,3$$

To implement the QPSK modulation, we only need to modulate the phase coefficients of the reflected harmonics to generate the appropriate Γ_*m*_(*t*). According to Equation (8), we can easily establish the mapping relationship between the duty cycle *D*, the time delay *t*_0_, and Γ_*m*_(*t*) = *Ae* ^*j*φ^. As inferred in Section 3, selecting the appropriate time delay *t*_0_ allows us to obtain the desired constellation set. For the +1st order harmonic, we only need to set the time delay *t*_0_ of the modulation signal to be 7*T*_0_/8, 5*T*_0_/8, 3*T*_0_/8, and *T*_0_/8 respectively, with the duty cycle *D* consistently maintained at 0.5. And hence, the phase states of the four reflected signals can satisfy the constellation set. For convenience, we introduce the covariate $\gamma_{D}^{t_{0}}$ to represent the combination of duty cycle *D* and time delay *t*_0_ as the basis for time coding. With this covariate, the set of constellation maps can be represented as $\left\{\gamma_{0.5}^{0.875T_{0}},\gamma_{0.5}^{0.625T_{0}},\gamma_{0.5}^{0.375T_{0}},\gamma_{0.5}^{0.125T_{0}}\right\}$. Additionally, taking into account the amplitude coefficient |*h*| and phase coefficient φ_0_ (induced by the difference of the modulation signal between two states) added to each order of harmonics, as well as the distortion and amplitude attenuation |*h_v_* | and the additional phase change |φ_*v*_| caused by STCM during transmission, the actually received constellation set should be $\left|h|\right|h_{v}|e^{j(\varphi_{0}+\varphi_{v})}\left\{e^{j0.25\pi },e^{j0.75\pi },e^{j1.25\pi },e^{j1.75\pi }\right\}/\pi$. In this case, the received constellation map is normalized and phase‐corrected to convert it to a standard constellation map.

To further demonstrate the potential of the designed STCM for next‐generation wireless communication systems, we have implemented higher‐order modulation of sub‐terahertz communication by utilizing the phase and amplitude modulation capabilities of the STCM for reflected harmonics. In this paper, we verify the QAM scheme by implementing the 16QAM. By applying similar methods, we can further realize 64QAM and 256QAM schemes. In the future, after optimizing the hardware structure, we may also achieve more advanced modulation schemes such as 1024QAM and 4096QAM. Our STCM holds significant promise for next‐generation wireless communication systems.

In line with the established communication theories and the earlier elaborations on FSK and QPSK modulation schemes, the constellation points for the standard 16QAM configuration are as follows

(11) $$\begin{array}{c} \begin{array}{c} \Gamma_{m}\left(t\right)\in M=\left\{\begin{array}{cccc} e^{j0.75\pi }, & \frac{\sqrt{5}}{3}e^{j0.6\pi }, & \frac{\sqrt{5}}{3}e^{j0.4\pi }, & e^{j0.25\pi },\\ \frac{\sqrt{5}}{3}e^{j0.9\pi }, & \frac{1}{3}e^{j0.75\pi }, & \frac{1}{3}e^{j0.25\pi }, & \frac{\sqrt{5}}{3}e^{j0.1\pi },\\ \frac{\sqrt{5}}{3}e^{-j0.9\pi }, & \frac{1}{3}e^{-j0.75\pi }, & \frac{1}{3}e^{-j0.25\pi }, & \frac{\sqrt{5}}{3}e^{-j0.1\pi },\\ e^{-j0.75\pi }, & \frac{\sqrt{5}}{3}e^{-j0.6\pi }, & \frac{\sqrt{5}}{3}e^{-j0.4\pi }, & e^{-j0.25\pi } \end{array}\right\}\\ \left|M\right|=16,m=0,1,\cdot \cdot \cdot ,14,15 \end{array} \end{array}$$

The standard constellation diagram reveals that 16QAM necessitates further modulation of the amplitude coefficient in addition to the phase coefficient modulation similar to QPSK. The set of constellation points represented by the parameter $\gamma_{D}^{t_{0}}$ can be expressed by

(12) $$\left\{\begin{array}{cccc} \gamma_{0.5}^{0.625T_{0}}, & \gamma_{0.268}^{0.7T_{0}}, & \gamma_{0.268}^{0.8T_{0}}, & \gamma_{0.5}^{0.875T_{0}},\\ \gamma_{0.268}^{0.55T_{0}}, & \gamma_{0.108}^{0.625T_{0}}, & \gamma_{0.108}^{0.875T_{0}}, & \gamma_{0.268}^{0.95T_{0}},\\ \gamma_{0.268}^{0.45T_{0}}, & \gamma_{0.108}^{0.375T_{0}}, & \gamma_{0.108}^{0.125T_{0}}, & \gamma_{0.268}^{0.05T_{0}},\\ \gamma_{0.5}^{0.375T_{0}}, & \gamma_{0.268}^{0.3T_{0}}, & \gamma_{0.268}^{0.2T_{0}}, & \gamma_{0.5}^{0.125T_{0}} \end{array}\right\}$$

For 16QAM, we perform normalization and phase correction in a manner similar to QPSK. Afterward, we further establish the mapping between data symbols, providing the foundation for utilizing STCM for information modulation as a transmitter (see Note S6, Supporting Information, for more details).

**Figure 5:**
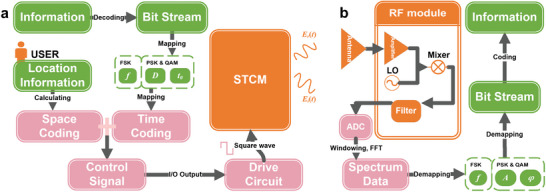
a,b) Transmitter and receiver of integrated communication transmitter and beam scanning system based on STCM.

To illustrate the application of our designed STCM in a communication system and showcase its ability to achieve independent control in space and time, as well as the way to integrate the communication transmitter and the beam scanning system, we have developed schematics as shown in **Figure** **5**. At the transmitter side (Figure 5a), the host computer decodes the information (e.g., images, videos, and other file types) into a binary bit stream for transmission to the baseband module. The bit stream is subsequently mapped to the corresponding time coding based on different modulation schemes. For BFSK, by mapping “0” to “*f*_1_” and “1” to “*f*_2_”, the time coding is similar to “*f*_1_ *f*_2_ *f*_2_ *f*_1_ *f*_2_⋅⋅⋅”. For QPSK, “00” is mapped to “$\gamma_{0.5}^{0.625T_{0}}$”, “01” is mapped to “$\gamma_{0.5}^{0.875T_{0}}$”, “10” is mapped to “$\gamma_{0.5}^{0.375T_{0}}$”, and “11” is mapped to “$\gamma_{0.5}^{0.125T_{0}}$”, with the time coding resembling “$\gamma_{0.5}^{0.375T_{0}}\gamma_{0.5}^{0.375T_{0}}\gamma_{0.5}^{0.625T_{0}}\gamma_{0.5}^{0.875T_{0}}\gamma_{0.5}^{0.125T_{0}}\cdots$”. For other higher‐order modulation schemes, such as MPSK and QAM, the mapping process is similar. Subsequently, the time coding is coupled to the space coding, derived from the user's position information relative to the STCM, to obtain the final control signal, which is output by the digital input‐output (IO) module of the FPGA. In this process, for BFSK, once the space coding of the +1st order harmonic is determined, its beam pointing is established, and the space coding of the −1st order harmonic is also determined, enabling independent control of time coding and space coding. For PSK and QAM, the duty cycle *D* independently controls the signal amplitude with time, while the signal phases switch according to the time delay *t*_0_ that varies over time. As the time coding of different columns of the signal is switched simultaneously, according to Equation (1), the phase gradient between different columns of the STCM remains stable, ensuring a stable beam pointing. To control the beam pointing, it is only necessary to adjust the initial space coding, and the time coding and space coding can be controlled independently. The output digital signal is then amplified by a drive circuit and applied to the STCM, which reflects an EM wave carrying digital information at the 1st order harmonic with a fixed beam direction. In this way, the STCM successfully implements the synthesis of a communication transmitter and a beam steering system.

At the receiver side (Figure 5b), the received EM wave is processed by an RF module with a superheterodyne structure, where the down‐conversion frequency is set to *f_c_*. The time‐domain signal, obtained from the analog‐to‐digital converter sampling, is converted to a frequency‐domain signal through a fast Fourier transform (FFT). For FSK, the amplitude at *f*_1_ = *f*_0_ and *f*_2_ = −*f*_0_ are detected, if *f*_1_ is larger, the demapping is “0”, and if *f*_2_ is larger, the demapping is “1”. For QPSK, QAM, and other modulation schemes, *A* and φ at “*f*_0_” are detected, and the bit stream is demapped according to the table in Note S6 (Supporting Information). Finally, the host computer reconstructs the transmitted file based on the obtained binary bit stream.

## 5 Experiments of the Sub‐Terahertz Wireless Transmitter Based on STCM

**Figure 6:**
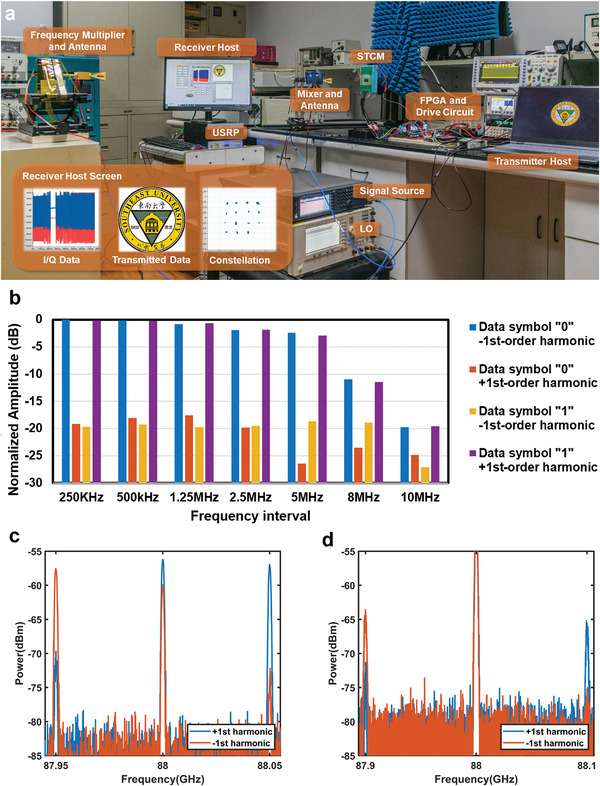
a) The experimental system established for sub‐terahertz wireless communication. b) The spectrum data obtained from FSK modulation at different frequency intervals. After updating the experiment equipment, the spectrum distribution at the frequency interval (c) *f*_0_ = 50 MHz and (d) *f*_0_ = 100 MHz.

In light of the provided description and the fabricated STCM, we constructed a sub‐terahertz wireless communication experiment system, as illustrated in **Figure** **6**a. We employed a microwave signal source (Keysight N5183B) connected to a frequency multiplier as the excitation source, generating the desired carrier wave at a fixed frequency via a linear polarization horn. Drawing from experimental results from Section 3, the carrier frequency was set at 88 GHz. A computer functioned as the transmitter host, with an FPGA module (Xilinx Kintex‐7) and a custom‐built drive circuit module employed to load the required control signals onto the STCM. Our system operates within the sub‐terahertz frequency range, significantly exceeding frequencies receivable by a universal software radio peripheral (USRP). As a result, the receiver side employs a superheterodyne architecture, down‐converting the received signals to the sub‐6 GHz via the RF module. In this experiment, a microwave signal source (Agilent E8257D) connected to a frequency multiplier serves as the local oscillator (LO) with an operating frequency of 86 GHz. The USRP (USRP X310) is configured to demodulate the down‐converted signal with a center frequency of 2 GHz, facilitating sampling, and baseband operation. Finally, the recovered transmitted bitstream is recoded into the corresponding file (in this experiment, an image of the Southeast University logo). This experiment demonstrates the efficacy of the communication system we devised (see Note S7, Supporting Information, for more details).

Using the experiment system, we set the STCM's space coding to direct the beam at −35^o^ and subsequently coupled the time coding. We successfully executed communications using the BFSK, QPSK, and 16QAM modulation schemes and measured the spectrum (BFSK) and constellation diagram (QPSK, 16QAM) at varying frequency intervals *f*_0_. Test results from Section 3 indicate that communications can be achieved at different beam angles. Figure 6b displays the measured spectrum at distinct frequency intervals. We observed that the amplitude difference of the ±1st order harmonics in the spectrum of data symbols “0” and “1” exceeds 15 dB at the majority of frequency intervals, indicating that our designed STCM transmitter is capable of high‐quality FSK communications. As the frequency interval *f*_0_ increases, the harmonic components of other frequencies emerge due to the distortion of signal produced by the FPGA output and drive circuit. Consequently, the amplitude of the anticipated 1st order harmonic component declines and the difference between the ±1st order harmonics within the same data symbol diminishes. At a 10 MHz frequency interval, the amplitude difference of the ±1st order harmonics in data symbol “0” is at least 5.18 dB, surpassing 3 dB and still enabling effective FSK communications. To ensure the corresponding harmonic frequency's power magnitude and minimize the truncation effects, the data symbol duration should be an integer multiple of the modulated signal period. In this case, we chose *T*′ = *T*_0_, which can achieve a communication rate of 10 Mbps.

To reach higher communication rates, we can upgrade the FPGA and drive circuit in the transmitter with two waveform generators (RIGOL DG5352), which produce four types of time‐delayed square wave signals at frequencies up to 100 MHz. Accordingly, the receiver requires a bandwidth of more than 200 MHz and a sampling rate in hundreds of MS/s, which cannot be supported by our existing USRP. Hence, we utilized a spectrum analyzer (Keysight N9040B) as the receiver. We performed the spectrum measurements at frequency intervals of 50 and 100 MHz, as illustrated in Figure 6c,d. It is observed that at the 100 MHz interval, the power intensity difference between the +1st and −1st harmonics in the data symbol “0” reaches 7.75 dB, which evidences that a communication rate up to 100 Mbps is definitely achievable while maintaining stable FSK communications. By upgrading the control devices of the transmitter, we notably enhance the system performance and conclude that the designed STCM can support higher communication rates, depending on the performance of the control module and the response rate of the varactor diodes.

**Figure 7:**
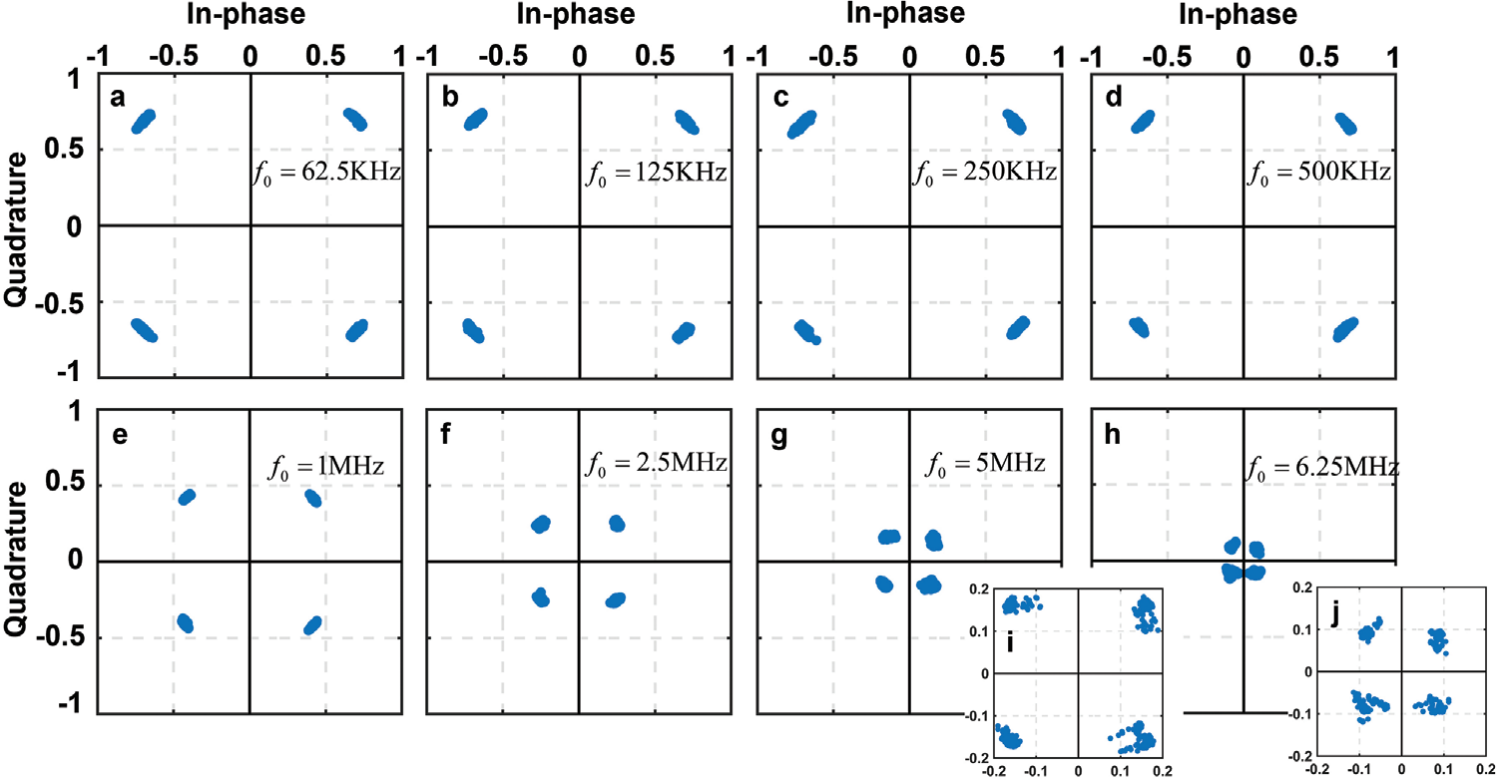
The constellation diagrams of the QPSK modulation in communications obtained at different frequency intervals.

**Figure 8:**
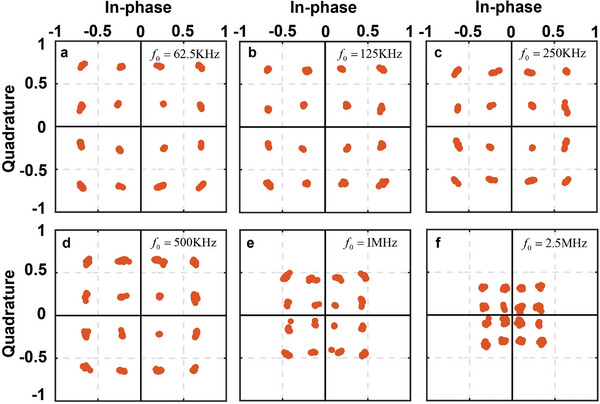
The constellation diagrams of the 16QAM modulation in the communications obtained at different frequency intervals.

For QPSK and QAM, constellation diagrams are employed to represent the relationship between their baseband signals, which are portrayed in a complex in‐phase/quadrature (I/Q) plane. **Figures** **7** and **8** display the measured constellation diagrams for the QPSK and 16QAM communications at various frequency intervals*f*_0_. The data are normalized to the maximum amplitude of all data in the diagram, and it is evident that the measured constellation diagrams closely align with the standard constellation diagram. As the frequency interval *f*_0_ increases, the measured amplitude value decreases while the phase is approximately accurate. Nevertheless, the Error Vector Magnitude (EVM) performance experiences a certain degree of deterioration. This is because, when the frequency interval increases, the frequency of the modulation signal generated by the FPGA and the drive circuit also increases. As the frequency gradually rises, FPGA and the drive circuit, we employed, can no longer accurately control the duty cycle of the signal loaded onto the STCM. Additionally, due to performance limitations, the USRP, we utilize, becomes under‐sampled, preventing us from modulation tests at higher frequency intervals.

In the experiment, QPSK operates at the frequency interval *f*_0_ = 6.25MHz, and 16QAM operates at the frequency interval *f*_0_ = 2.5MHz. Considering the data symbol duration *T*′ = *T*_0_, their communication rates can reach 12.5 and 10 Mbps, respectively. As we mentioned in the section of FSK modulation communication test, this is not the limit of the communication rate that the proposed STCM can attain, and higher communication rates become available with the improvements and upgrades of the control devices and experiment equipment.

**Figure 9:**
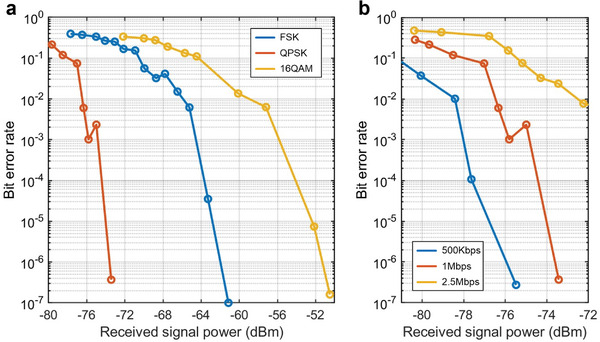
a) BER test results at 1 Mbps communication rate using FSK, QPSK, and 16QAM modulation schemes, respectively. b) BER test results of QPSK at different communication rates.

To evaluate the performance of the STCM‐based sub‐terahertz wireless communications, we examined the bit error rate (BER) of the system under varying received signal powers, modulation schemes, and communication rates, as depicted in **Figure** **9**. The results in Figure 9a indicate that, for a fixed communication rate of 1 Mbps, the QPSK scheme shows a lower BER than the FSK modulation scheme. This is attributable to its superior noise resistance and spectral efficiency. However, the increased symbol density in the 16QAM scheme makes it more prone to noise‐induced errors, channel impairments, and interference, resulting in a higher BER. Moreover, take the QPSK modulation scheme as an example, Figure 9b shows that for the same modulation scheme, increasing the communication rate will lead to an escalated BER. This arises from the heightened system vulnerability to noise, amplified impact of interference and channel impairment, and increased inter‐symbol interference. As the communication rate increases, the distortion of the square‐wave modulation signal generated by the FPGA and the driving circuit results in a higher BER. Collectively, these factors contribute to an increased BER for the entire communication system. We wish to emphasize that the proposed STCM is not the primary obstacle to reduce the BER. In the future, we anticipate that through improving and upgrading the control device and experiment equipment, the BER can be further reduced.

**Figure 10:**
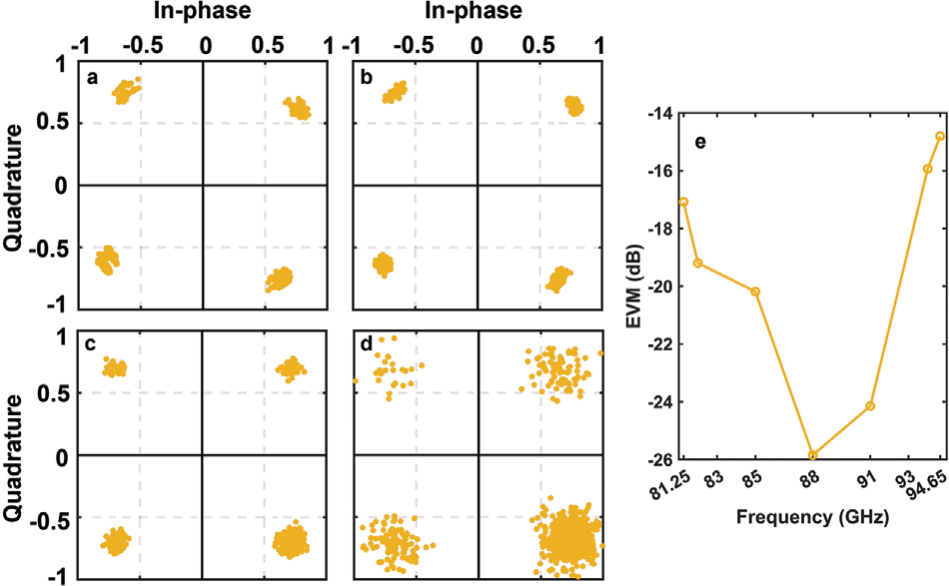
The constellation diagram of QPSK modulation in communication, obtained at the communication rate of 500 Kbps, obtained at operating frequencies of a) 82, b) 85, c) 91, and d) 94 GHz, respectively. e) The EVM experiment results of the QPSK constellation with a communication rate of 500 Kbps.

As the frequency varies, the reflection amplitude and phase of the STCM unit may deviate from perfection. However, based on Equation (8), this deviation will primarily result in reducing the harmonic reflection efficiency, rather than affecting the relative control relationship between the time delay and duty cycle of the square‐wave control signal on the phase and amplitude of the reflected harmonic. Therefore, the proposed STCM and modulation method exhibit robust controls of the reflected harmonics, we anticipate that the STCM can achieve communication functionality across a broader frequency band. We conducted the experiment on the QPSK modulation scheme at a communication rate of 500 Kbps and found that the STCM can operate effectively from 81.25 to 94.65 GHz. We selected four frequencies of 82, 85, 91, and 94 GHz to construct the normalized constellation diagrams, as presented in **Figure** **10**a–d. Maintaining the transmission power, we observed that the BER obtained in the experiment was below 10^−7^ over the entire frequency range. The EVM is less than −25 dB at 88 GHz and deteriorates beyond 88 GHz, but it still preserves excellent performance. As demonstrated in Figure 10e, the EVM is −17.08 dB at 81.25 GHz and −14.81 dB at 94.65 GHz. Indeed, a sharp decline in both BER and EVM performance appears outside of the frequency range, which can probably be ascribed to the bandwidth performance of custom‐made RF devices. Therefore, a wider operation bandwidth of the STCM‐based sub‐terahertz wireless communication system can be expected. Furthermore, we remark that for our communication system, the dynamic power consumption of the designed STCM array is ≈200 mW, which indicates the potential application in green communications.

## 6 Conclusion

We have proposed an STCM designed for sub‐terahertz operation. Leveraging our unique EM manipulation mechanism and coding method, we successfully implemented various modulation schemes, including FSK, PSK, and QAM, over a wide bandwidth. Our communication system is capable of realizing effective beam scanning for user tracking within ±45^o^. This showcases the impressive information modulation and beam steering capabilities of our designed STCM and EM manipulation mechanism in the sub‐terahertz frequencies, effectively achieving the integration of information modulation and beam steering. Compared with the conventional wireless communication transmitter architectures applied to the sub‐terahertz band, our STCM transmitter provides an alternative with low complexity, low cost, low power, and low heat dissipation. We believe that our design approach, EM manipulation mechanism, coding method, and system architecture can offer a viable, innovative solution for future industry applications in next‐generation wireless communication systems targeting sub‐terahertz and even terahertz band communications.

## Conflict of Interest

The authors declare no conflict of interest.

## Supporting information

Supporting Information

Supplemental Video 1

## Data Availability

The data that support the findings of this study are available from the corresponding author upon reasonable request.
