# Supplementary material for: Toward Sub‐Terahertz: Space‐Time Coding Metasurface Transmitter for Wideband Wireless Communications
Source: Adv Sci (Weinh). 2023 Aug 8;10(29):2304278. doi: 10.1002/advs.202304278 (PMC10582441; doi:10.1002/advs.202304278)
Supplement: Supplementary file 1 — Supporting Information [file ADVS-10-2304278-s001.pdf]

ADVANCED SCIENCE
Open Access

Supporting Information

for *Adv. Sci.*, DOI 10.1002/advs.202304278

Toward Sub-Terahertz: Space-Time Coding Metasurface Transmitter for Wideband Wireless Communications

Yujie Liu, Yu Wang, Xiaojian Fu, Lei Shi, Fei Yang, Jiang Luo, Qun Yan Zhou, Yuan Fu, Qi Chen, Jun Yan Dai, Lei Zhang, Qiang Cheng and Tie Jun Cui**

Supporting Information

Towards Sub-Terahertz: Space-Time Coding Metasurface Transmitter for Wideband Wireless Communications

Yujie Liu, Yu Wang, Xiaojian Fu, Lei Shi, Fei Yang, Jiang Luo, Qun Yan Zhou, Yuan Fu, Qi Chen, Jun Yan Dai, Lei Zhang, Qiang Cheng, Tie Jun Cui**

Supplementary Note 1: Equivalent Circuit of STCM unit

Figure 2(b) presents the equivalent circuit diagram of the STCM unit structure, obtained through the metasurface equivalent circuit analysis method. High-frequency current flowing through the metal structure generates a quasi-static magnetic field, allowing representation of the symmetrical metal patches as inductors L_1 and L_2. Considering that the two metal patches are connected to DC and GND, respectively, L_1 and L_2 are not equal. The coupling capacitance between adjacent units can be represented by C. The varactor diode can be modeled as a circuit containing a parasitic capacitor C_P in parallel with a series circuit consisting of a resistor R_S, a parasitic inductor L_S, and a variable capacitor $C_\mathrm{J}(V)$, where $C_\mathrm{J}(V)$ depends on the bias voltage across the varactor diode. The varactor diode is connected to a symmetrical metal patch, which can be regarded as the varactor diode's equivalent circuit in series with L_1 and L_2. Consequently, the effective impedance of the STCM's equivalent circuit at varying voltages can be expressed as

$$Z_\mathrm{C}(\omega) = j\omega L_1 + j\omega L_2 + \cfrac{1}{j\omega C_\mathrm{P} + \cfrac{1}{R_\mathrm{S} + j\omega L_\mathrm{S} + \cfrac{1}{j\omega C_\mathrm{J}(\mathrm{V})}}} \tag{S1}$$

For a free-space plane wave with the electric field direction oriented along the y-axis, as depicted in Figure 2(b), the reflection coefficient observed from the incident port can be described by

$$\Gamma = r(\omega) = \frac{Z_\mathrm{C}(\omega) - Z_0}{Z_\mathrm{C}(\omega) + Z_0} = |R| e^{j\omega\phi} \tag{S2}$$

where $Z_0 = 377\Omega$ represents the free-space impedance, and Z_C denotes the variable effective impedance, which depends on $C_J(V)$. To achieve a phase difference of π between the two reflected phases, the reflection coefficients Γ_0 and Γ_1 for the two states must satisfy

$$\angle(\Gamma_1/\Gamma_0) = \pm\pi \tag{S3}$$

In other words, the bias voltage across the varactor diode should be configured to ensure a phase difference of π between the reflections of the two states. This condition establishes the distinction between code "0" and code "1".

Supplementary Note 2: Details of STCM Design Using Varactor Diodes in Sub-Terahertz

The MA46H146 varactor diode selected for the proposed STCM is a GaAs flip-chip varactor diode characterized by extremely low total capacitance ($< 0.06\text{pF}$) and a high Q value ($> 15\text{K}$). Capable of withstanding a reverse bias voltage of up to 25V, its capacitance varies from 0.063pF to 0.03pF. The capacitance-voltage (C-V) curve is illustrated in **Figure S1(a)**. The STCM unit structure is simulated using CST Microwave Studio, with the varactor diode's S-parameters obtained through Advanced Design System simulation imported into the lumped element module. This module has dimensions of 665μm in length and 340μm in width. The S-parameters of the varactor diode at various bias voltages are imported to simulate the STCM's performance under different DC signals. By doing so, we investigate the operation of the STCM under various DC signals. The S11 amplitude and phase of the STCM unit, when the varactor diode is loaded with 0V, 4V, 8V, 16V, and 24V bias voltages, are illustrated in Figures S1(b) and (c), respectively. It is evident that the resonant frequency shows a blue shift as the voltage increases. The resonant point changes more rapidly at low voltages and tends to flatten out at higher voltages, a trend that aligns with the C-V curve shown in **Figure S1(a)**. At low voltages, the capacitance experiences significant changes with voltage, while at high voltages, the capacitance variations tend to be more gradual. At 94 GHz, the STCM's amplitude values are approximately the same when loaded with 0V and 24V bias voltages. Moreover, the phase difference between the two states is π.

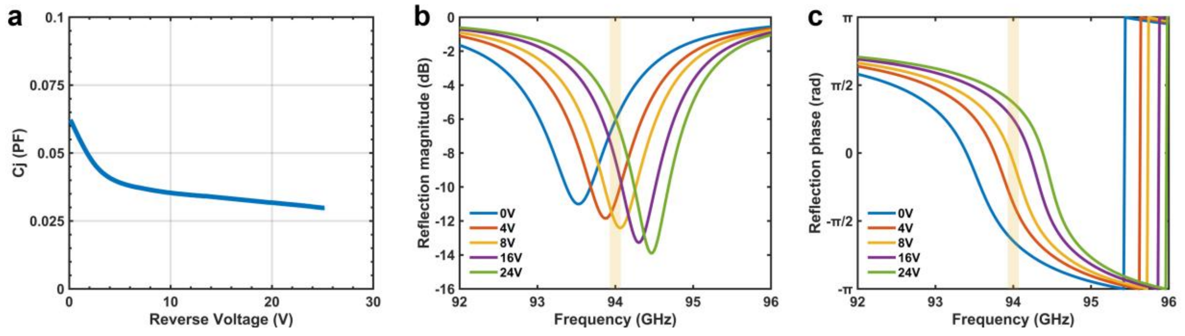

Figure S1. (a) C-V plot for the MA46H146 varactor diode. Reflection wave (b) Amplitude and (c) phase simulation results of the STCM under 0V, 4V, 8V, 16V, and 24V voltage states.

Supplementary Note 3: Simulation of STCM Beam Scanning with Space-coding

According to the method described in Section 2, we selected 6 different reflection angles ranging from 0-55° to calculate the coding states and simulated the far-field scattering patterns, with the results shown in **Figure S2**. The findings reveal that the far-field main beam exhibits left-right symmetry, with the angle essentially aligning with the design angle. The designed STCM is capable of achieving beam scanning within a ±55° range.

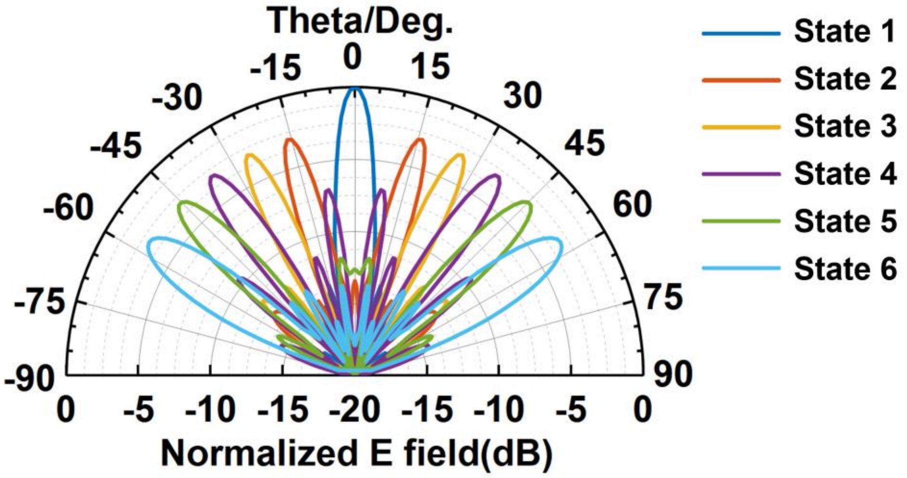

Figure S2. Simulation results of far-field scattering patterns for STCM employing various space coding states. Coding state 1 (all 0), state 2 (1100001111000011), state 3 (1100111001110011), state 4 (1101100110011011), state 5 (1001001001001001), and state 6 (1011010110101101) correspond to reflection angles 0°, 15°, 25°, 35°, 45°, and 55°, respectively.

Supplementary Note 4: Experimental Setup for Manipulation of Harmonics by STCM

In order to generate square wave modulation signals for each subarray, we designed an FPGA control module based on the Xilinx Kintex-7, which is connected to the drive circuit. This allows for real-time modification of the coding states. The sample and experiment environment are depicted in **Figure S3**. Given that the sample operates in the sub-terahertz, which is beyond the typical operating frequency range of signal sources and spectrometers, a frequency multiplier is required, and a superheterodyne structure is employed at the receiver side. The transmitter side utilizes a frequency multiplier to generate a signal at 88 GHz. At the receiver side, an RF module downconverts the received signal to 2 GHz, with a LO frequency of 86 GHz. The transmitter horn is positioned at the far end to provide a vertically incident, horizontally polarization plane wave, while the receiver horn is mounted on the extension arm of the turntable to measure the far-field scattering intensity in the two-dimensional plane surrounding the sample.

The frequency shift from 94 GHz to 88 GHz can be primarily ascribed to the parasitic capacitance generated during the surface mounting process and EM coupling among the units. Since the unit size of the sample is small, errors introduced by the mounting process have a considerable impact. Moreover, errors of photolithography processing and inherent tolerances in the device’s dimensions are significant relative to the unit size, leading to discrepancies in the actual fabrication that may also result in a shift in the resonance point.

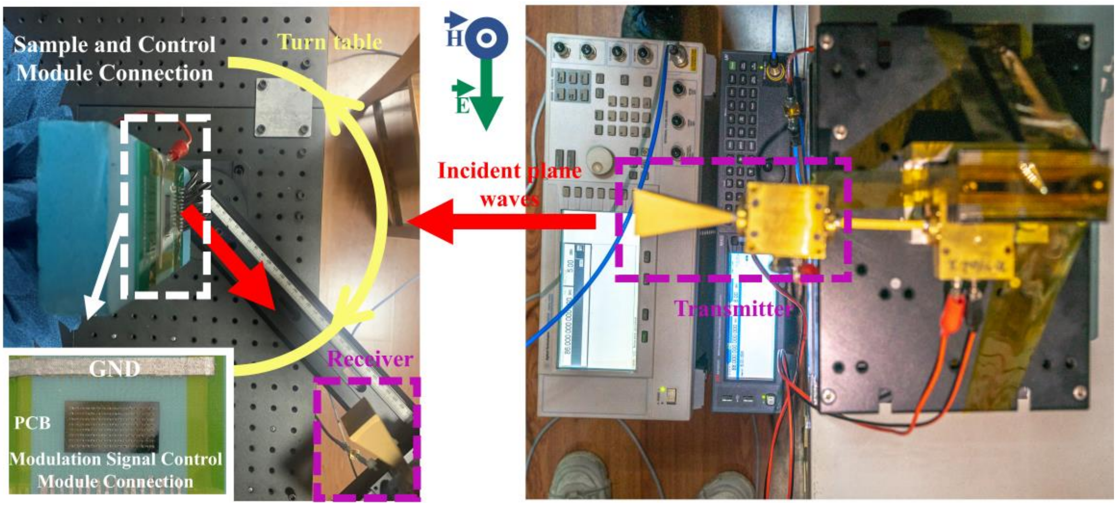

Figure S3. Processing sample and experimental test environment.

Supplementary Note 5: FSK Communication Using ±1st Order Harmonics

According to **Equation (9)**, when the STCM is loaded with a duty cycle of 0.5, the time delays of 0, $-T_0/4$, $-T_0/2$, and $-3T_0/4$ are coded as "0", "1", "2" and "3" modulation signals, respectively. For the +1st harmonic, the corresponding phases are φ_0, $\varphi_0+\pi/2$, $\varphi_0+\pi$, and $\varphi_0+3\pi/2$. For the -1st harmonic, the phases are φ_0, $\varphi_0-\pi/2$, $\varphi_0-\pi$, and $\varphi_0-3\pi/2$. This results in an exact reversal of the phase gradient between the same space coding. According to **Equation (1)**, the reflected harmonics exhibits left-right symmetry for the same coding states. To verify this, we calculated and measured the -1st order harmonics using the same space coding as shown in Figure 4. The calculated results in **Figure S4(a)** demonstrate perfect symmetry with those in Figure 4(a), and the experimental results in **Figure S4(b)** and Figure 4(b) are also fundamentally symmetric. Consequently, we can successfully achieve FSK communication using both ±1st order harmonics.

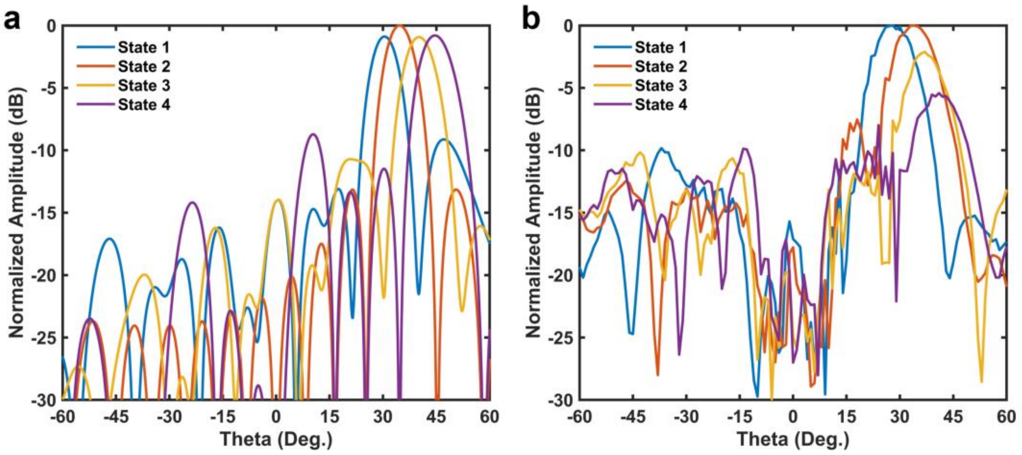

Figure S4. (a) Calculation and (b) Experiment of beam deflection achieved by varying the square wave control signal time delay to modulate the -1st order harmonic phase, resulting in deflection angles of 30°, 35°, 40°, and 45°, respectively.

Supplementary Note 6: Mapping of Data Symbols to Modulation Signals

According to **Equation (8), (9), (10), and (11)**, we can accurately establish the respective mapping relationship between QPSK and 16QAM data symbols and modulation signals, as illustrated in Table S1 and S2. While this paper only presents information related to QPSK and 16QAM, more complex modulation schemes such as 8PSK, 64QAM, 128QAM, and even 256QAM can be obtained using a similar approach.

Table S1. Mapping relationships between $\Gamma_m(t)$, $\gamma_D^{t_0}$ and data symbols for QPSK

$\Gamma_m(t)$	$e^{j\frac{\pi}{4}}$	$e^{j\frac{3\pi}{4}}$	$e^{j\frac{5\pi}{4}}$	$e^{j\frac{7\pi}{4}}$
$\gamma_D^{t_0}$	$\gamma_{0.5}^{7T_0/8}$	$\gamma_{0.5}^{5T_0/8}$	$\gamma_{0.5}^{3T_0/8}$	$\gamma_{0.5}^{T_0/8}$
Data Symbol	01	00	10	11

Table S2. Mapping relationships between $\Gamma_m(t)$, $\gamma_D^{t_0}$ and data symbols for 16QAM

$\Gamma_m(t)$	$\mathrm{e}^{0.75\pi}$	$\frac{\sqrt{5}}{3}\mathrm{e}^{0.6\pi}$	$\frac{\sqrt{5}}{3}\mathrm{e}^{0.4\pi}$	$\mathrm{e}^{0.25\pi}$
$\gamma_D^{t_0}$	$\gamma_{0.5}^{0.625T_0}$	$\gamma_{0.268}^{0.7T_0}$	$\gamma_{0.268}^{0.8T_0}$	$\gamma_{0.5}^{0.875T_0}$
Data Symbol	1011	1001	1110	1111
$\Gamma_m(t)$	$\frac{\sqrt{5}}{3}\mathrm{e}^{0.9\pi}$	$\frac{1}{3}\mathrm{e}^{0.75\pi}$	$\frac{1}{3}\mathrm{e}^{0.25\pi}$	$\frac{\sqrt{5}}{3}\mathrm{e}^{0.1\pi}$
$\gamma_D^{t_0}$	$\gamma_{0.268}^{0.55T_0}$	$\gamma_{0.108}^{0.625T_0}$	$\gamma_{0.108}^{0.875T_0}$	$\gamma_{0.268}^{0.95T_0}$
Data Symbol	1010	1000	1100	1101
$\Gamma_m(t)$	$\frac{\sqrt{5}}{3}\mathrm{e}^{-0.9\pi}$	$\frac{1}{3}\mathrm{e}^{-0.75\pi}$	$\frac{1}{3}\mathrm{e}^{-0.25\pi}$	$\frac{\sqrt{5}}{3}\mathrm{e}^{-0.1\pi}$
$\gamma_D^{t_0}$	$\gamma_{0.268}^{0.45T_0}$	$\gamma_{0.108}^{0.375T_0}$	$\gamma_{0.108}^{0.125T_0}$	$\gamma_{0.268}^{0.05T_0}$
Data Symbol	0001	0000	0100	0110
$\Gamma_m(t)$	$\mathrm{e}^{-0.75\pi}$	$\frac{\sqrt{5}}{3}\mathrm{e}^{-0.6\pi}$	$\frac{\sqrt{5}}{3}\mathrm{e}^{-0.4\pi}$	$\mathrm{e}^{-0.25\pi}$
$\gamma_D^{t_0}$	$\gamma_{0.5}^{0.375T_0}$	$\gamma_{0.268}^{0.3T_0}$	$\gamma_{0.268}^{0.2T_0}$	$\gamma_{0.5}^{0.125T_0}$
Data Symbol	0011	0010	0101	0111

Supplementary Note 7: Supplementary Video

In this video, we showcase a sub-terahertz wireless communication system based on our designed STCM transmitter. In the demonstration, the carrier frequency is 88GHz, and the space-time coding metasurface transmitter cyclically sends the logo of Southeast University with a fixed space coding state. The receiver orientates with a deflection angle of -35°. The down-converted signal is sampled and processed by the USRP before being displayed in real-time on the receiver host’s screen. To enhance the visibility, we have enlarged the content on the screen and displayed it in the lower left corner.

Our tests include FSK modulation scheme with rates of 1Mbps and 3.125Mbps, QPSK modulation scheme with rates of 2.5Mbps and 5Mbps, and 16QAM with a 2Mbps rate. When we blocked the RF link with a metal plate, the signal-to-noise ratio of the received data decreased, causing distortion in both the constellation diagram and the recovered image. However, when we removed the metal plate, the received data returned to normal immediately.

It needs to be added that the tested communication rate is not the limit that the sub-terahertz wireless communication based on the STCM can achieve. With the improvement of the control module and experiment equipment, we can measure a higher communication rate. Overall, we remark that the sub-terahertz STCM developed in this work demonstrates the significant potential for constructing the novel-architecture wireless communication systems.
